# Mental Suggestion

**Published:** 1891-11

**Authors:** 


MENTAL SUGGESTION. By Dr. J. Ochorowicz, sometime Professor Extraordinary of Psychology and Natural Philosophy in the University of Lemberg. Four double numbers of the *Humboldt Library*. Price, $1.20. New York: The Humboldt Publishing Co., 19 Astor Place.

Much is nowadays said and written about *Hypnotism:* the more ancient term *Animal Magnetism* is not often mentioned. It is the common belief that whatever of truth there was in the doctrines of Mesmer, Puységur, and the rest of the "animal magnetizers" is comprised under the scientific term "hypnotism," and that the modern school of Charcot, and the school of "suggestionists" at Nancy, France, represent the highest attainment in the science and art once studied and practiced by Mesmer and Puységur, and later investigated by Braid, of Manchester. But here is an author who maintains that hypnotism and animal magnetism, though they have certain superficial resemblances, are radically different from each other in their phenomena and in the modes of their production, and that the facts of magnetism are incomparably the more wonderful and the more worthy of scientific study. The title of the work, "*Mental* Suggestion," well marks the difference between hypnotism and magnetism: in hypnotism *mental* suggestion is not to be thought of, but that it exists in animal magnetism is the task of this author to prove.

The author is in every way competent to treat the subject: he is a learned physiologist and physicist, as well as a psychologist—and he has studied the matter experimentally for years. He has mastered all the literature of hypnotism and animal magnetism: his book contains an enormous amount of information nowhere else accessible outside of the greatest libraries. Just because Ochorowicz first explored the ground thoroughly on his own account and then sifted the bibliography of magnetism, he is able to estimate the true value of the work of prior experimenters and prior students and theorizers.

It is simple truth to say that no student of human psychology can afford to neglect this most able and brilliant treatise—a work original in its methods as in its points of view, and possessing moreover all the charms of a consummate literary style—in other words, consummate simplicity and clearness of expression. It is unquestionably the completest work on magnetism and hypnotism ever written: no author so well equipped for the discussion of the question ever attempted it before.

